# Effects of Advanced Platelet-Rich Fibrin on Bone Healing in the Treatment of Canine Appendicular Fractures

**DOI:** 10.3390/ani16081276

**Published:** 2026-04-21

**Authors:** Ravisa Warin, Preeyanat Vongchan, Witaya Suriyasathaporn, Ratchadaporn Boripun, Kanawee Warrit, Luddawon Somrup, Kittidaj Tanongpitchayes, Pimnipa Jieraviriyapun, Wanna Suriyasathaporn

**Affiliations:** 1 Akkhraratchakumari Veterinary College, Walailak University, Nakhon Si Thammarat 80160, Thailand; ravisa.wa@wu.ac.th (R.W.); ratchadaporn.bo@wu.ac.th (R.B.); 2 Faculty of Veterinary Medicine, Chiang Mai University, Chiang Mai 50100, Thailand; suriyasathaporn.witaya.y3@f.mail.nagoya-u.ac.jp (W.S.); kanawee.w@cmu.ac.th (K.W.); pimnipa_jie@cmu.ac.th (P.J.); 3 Department of Medical Technology, Faculty of Associated Medical Sciences, Chiang Mai University, Chiang Mai 50200, Thailand; preeyanat.v@cmu.ac.th; 4 Research Center of Producing and Development of Products and Innovations for Animal Health and Production, Chiang Mai University, Chiang Mai 50100, Thailand; 5 Asian Satellite Campuses Institute-Oversea Campus, Nagoya University, Nagoya 464-8601, Japan; 6 Northern Regional Veterinary Healthcare and Learning Center, Faculty of Veterinary Medicine, Chiang Mai University, Chiang Mai 50100, Thailand; luddawan.s@cmu.ac.th; 7 Faculty of Veterinary Science, Prince of Songkhla University, SongKhla 90110, Thailand; kittidaj.t@psu.ac.th

**Keywords:** advanced platelet-rich fibrin, dogs, traumatic bone fracture, bone healing

## Abstract

Although internal fixation and surgical approaches promote fracture healing, some outcomes remain unsatisfactory. Advanced platelet-rich fibrin (A-PRF) has been shown to provide more growth factors, and in vitro cell proliferation has not been evaluated for treating bone fractures in veterinary medicine. The purpose of this study was to evaluate the bone-healing activity of A-PRF in traumatic canine fractures. Twelve dogs with single radius–ulna or tibia–fibula fractures were randomly assigned to two groups: a control group and an A-PRF group. Both groups were treated with a locking compression plate and screws and received pain control. Post-operatively, dogs were evaluated for serum C-reactive protein (CRP) levels and post-operative pain scores on days 1, 3, and 7. Lameness and weight-bearing scores were evaluated on days 1, 3, 7, 14, 30, and 60. Bone healing was assessed at 2 weeks, 1 month, and 2 months using calculated relative bone density (%). Compared with the control, the A-PRF group showed higher bone density at 2 months and lower lameness at 14 days post-operatively. Although the CRP level, an inflammation response marker, was higher in the A-PRF group within one day. No significant difference in pain score was observed. In conclusion, A-PRF serves as an effective adjunctive therapy for promoting bone healing when treating canine appendicular fractures with surgical internal fixation.

## Simple Summary

Although surgical intervention is generally effective in promoting fracture healing, some outcomes remain unsatisfactory. Advanced platelet-rich fibrin (A-PRF), which has been shown to improve bone healing in vitro, has not been evaluated for clinical use in veterinary medicine. Thus, this study aimed to evaluate the bone-healing activity of A-PRF in traumatic canine fractures. Compared with the control, A-PRF showed progressive improvements in lameness and weight-bearing scores after 14 days post-surgery. Furthermore, higher relative bone density at 2 months was observed in A-PRF using radiography. CRP level, an inflammation response marker, was higher in A-PRF over a short period. No significant difference in pain score was observed. In conclusion, A-PRF offers an adjunctive therapy for promoting bone healing when treating canine appendicular fractures with surgical internal fixation, including increased bone density and improved limb functionality.

## 1. Introduction

Traumatic fractures of the appendicular bones constitute nearly 30 percent of musculoskeletal disorders in small animal patients [1]. Radius–ulna and tibia–fibula fractures are reported to be the most difficult to treat, often failing to regain their original properties despite receiving appropriate and long-term treatments [2]. It is crucial to establish an effective treatment strategy to shorten recovery time and improve bone healing. In general, successful bone regeneration management includes fracture stabilization, revascularization, and reduced infection [3]. Effective mechanical stabilization is essential for bone tissue formation, as it is linked to various healing processes [4]. For example, research indicates that mechanical strain exceeding 10% can compromise bone stabilization, leading to nonunion or delayed healing [5,6]. Angiogenesis is also crucial in the fracture-healing process, as it provides essential cells from nearby tissues and delivers nutrients and oxygen to the damaged area, thereby contributing to delayed fracture healing when angiogenesis is insufficient [7]. In long-bone fracture repair, various internal fixation methods have been evaluated to minimize mechanical strain and preserve periosteal vasculature; however, the outcomes remain unsatisfactory [8,9,10,11].

The most promising methods to promote bone healing include axial micromovement, electromagnetic stimulation, direct electrical currents, and administering growth factors and parathyroid hormones [12]. Activated platelets secrete biological materials, including growth factors such as platelet-derived growth factors (PDGFs), vascular endothelial growth factors (VEGFs), and transforming growth factors (TGFs). These factors play a vital role in stimulating angiogenesis and cell proliferation, which are essential for promoting bone regeneration [13,14]. The initial preparation of platelet concentrate, called platelet-rich plasma (PRP), has been used to enhance bone regeneration in canines [15,16]. The results regarding PRP’s functional outcomes are controversial. Preclinical studies on animals have shown an overall positive effect of PRP on osteoblast-like cells in vitro and on bone healing. However, in clinical cases, animals showed no significant improvement in the fracture-healing rate [15,16]. Despite its widespread use, nearly all PRP preparations are complex and expensive [17].

While PRP was a pioneer in regenerative medicine, platelet-rich fibrin (PRF) is widely considered “second-generation” because it overcomes several biological and chemical limitations of its predecessor. PRF has been shown to exhibit antimicrobial activity against some bacteria [18]. Studies on the application of PRF have demonstrated its ability to promote bone regeneration both in vitro and in vivo [19,20]. Some limitations of PRF in regenerative medicine led to the development of a new technique for obtaining PRF, called advanced PRF (A-PRF) [21], to achieve higher, more sustained growth factor release than standard PRF and PRP [22]. While standard PRF is centrifuged at 2700 rpm for 12 min, A-PRF is centrifuged at a slower speed for a longer time (1500 rpm, 14 min). In clinical studies, PRF has excellent osteogenic potential and has been widely used in bone tissue engineering and dentistry [23]. In orthopedic veterinary medicine, PRF has been reported to be effective in experimental acute articular cartilage injuries in dogs’ knees [24,25]. In veterinary clinical studies, radius–ulna and tibia–fibula fractures are common in dogs and cats, and treatment outcomes remain unsatisfactory, as described above. The healing process for fractures involves multiple stages, during which various assessments—such as physical examinations for pain and weight-bearing capabilities, along with radiological evaluations—are utilized [26]. However, no research has evaluated the effect of A-PRF, an advanced approach to producing PRF, on canine bone healing, especially in clinical practice. We hypothesized that A-PRF would accelerate bone fracture healing more effectively than a control group by providing sustained growth factor release and a stable fibrin scaffold to support cell migration. Therefore, this study aimed to determine the efficacy of canine A-PRF in promoting the bone-healing process in radius–ulna and tibia–fibula fractures. A physical examination and blood collection results, including pain score, lameness score, weight-bearing score, serum C-reactive protein (CRP) level (a protein produced by the liver in response to inflammation, infection, or tissue damage), and radiographic bone density, were evaluated.

## 2. Materials and Methods

The animal use protocol complied with the Animal Care and Use Committee, Faculty of Veterinary Medicine, Chiang Mai University, Thailand (FVM-CMU-ICUC Ref. No. S27/2563). The design was a prospective randomized exploratory clinical study. All dogs participated with their owners’ written informed consent. Owners were thoroughly apprised of the experimental procedures and had the right to withdraw their dog at any time. The sample size was calculated from the study of reduction in relative centrifugation force within injectable platelet-rich fibrin (PRF) concentrates advances patients’ own inflammatory cells, platelets and growth factors: the first introduction to the low speed centrifugation concept using G-power program at the alpha error was 5% and the power was 80% [27].

Dogs admitted to Chiang Mai University Small Animal Teaching Hospital with single tibia–fibula or radius–ulna fractures diagnosed radiographically were evaluated for overall health status according to the hospital’s routine protocols, including physical examinations, hematology (complete blood count), and blood biochemistry (total protein, albumin, blood urea nitrogen, creatinine, alanine aminotransferase, aspartate aminotransferase, and alkaline phosphatase). For animal health status, they were classified according to the American Society of Anesthesiologists (ASA) Physical Status Classification System [28]. Animals with traumatic ASA scores exceeding II, another concurrent disease, a history of antiplatelet or anticoagulant medication, and other orthopedic disorders, including open fracture, hip dysplasia, hip luxation, and cranial cruciate ligament rupture, were excluded from the study.

For each case, sex, breed, age (month), body weight (kg), bone fracture location, and fracture pattern data were collected. Pairs of patients were matched based on age, weight, and clinical signs and were subsequently randomly assigned to treatment and control groups. For the treatment group, a single A-PRF membrane was placed on the fracture line of the tibiofibular and the radioulnar bones at the time of surgery. A-PRF was prepared according to a previously published protocol [29]. Briefly, ten milliliters of whole blood without anticoagulant were centrifuged at 100× *g* for 14 min. After centrifugation, a fibrin clot formed in the middle of the tube between the red corpuscles at the bottom, and acellular plasma at the top was collected and frozen at −80 °C. All included animals received surgical treatment from experienced surgeons (LS and KT) using open reduction and internal fixation according to AO/ASIF principles [30] and the standard care protocol of Chiang Mai University Small Animal Teaching Hospital. The implants used were a locking compression plate and cortical screws. A medial approach was performed to place the locking compression plate over the fracture line. Cortical screws were used for at least 3 screws/fragment. Bacterial culture and drug sensitivity were performed using automated identification systems (VITEK^®^ 2 COMPACT, bioMérieux, Marcy l’Etoile, France) to ensure all cases were aseptic fractures. Any septic fracture was excluded. All dogs were administered amoxicillin–clavulanic acid at 13.75 mg/kg for 7 days as an antimicrobial. An analgesia protocol was used, including premedication with morphine 0.3 mg/kg, incisional anesthesia with bupivacaine 1 mg/kg, a fentanyl transdermal patch 2.2 µg/kg for 72 h post-operation, and Carprofen 4 mg/kg/day for 5 days post-operation. Cage rest was performed until bone union progression was radiographically presented and normal limb function was observed. Post-operatively, all dogs were evaluated for inflammation, pain, limb function, and radiography.

An assessment of inflammation was performed using serum C-reactive protein concentration. On days 1, 3, and 7 after surgery, 1 mL of blood was collected into a plain serum collection tube. Then, the serum C-reactive protein level was measured using a commercial fluorescent immunoassay test kit (Vcheck Canine CRP 2.0, Bionote USA Inc., Big Lake, MN, USA) and analyzer (Vcheck V200, Bionote USA Inc., Minnesota, USA) according to the manufacturer’s instructions. Based on the limit of detection of 10 mg/L, C-reactive protein levels below the LOD were set to 10 mg/L. Pain level was assessed using the Glasgow Composite Measure Pain Scales-Short Form (CMPS-SF) [31]. On days 1, 3, and 7 post-operatively, the researcher (RW) was blinded to the dog’s group to assess pain using the CMPS-SF form. Section B of the form was not completed due to mobility issues. Therefore, the total score of the CMPS-SF was 20. At 1, 3, 7, and 14 days, as well as at 1 month and 2 months post-operation, all dogs were blind-examined by a researcher (RW) for the lameness score (0 = no lameness; 4 = non-weight-bearing lameness) [32] and the weight-bearing score based on willingness to bear weight on the affected limb while standing (1 = typical weight bearing; 5 = no weight bearing), as well as willingness to lift the contralateral limb (1 = full willingness; 5 = no willingness to lift the limb) [33].

To ensure the reliability of the mean gray value (MGV) measurements, all radiographs from the same dogs at all times were obtained using the same digital X-ray unit with a fixed exposure protocol. At 14 days, 1 month, and 2 months after surgery, craniocaudal (CC) and mediolateral (LL) radiographic images of each individual were obtained and imported into ImageJ software (Version 1.44p, Wayne Rasband, National Institute of Health, Maryland, USA) to measure the mean gray value using the previously published protocol in [34], with slight modifications. Briefly, relative bone density was calculated using the following formula:

$$Relative\;bone\;density\;=\;\frac{Mean\;gray\;value\;of\;the\;defect\;region}{Mean\;gray\;value\;of\;the\;surrounding\;bone}$$

For accurate and reproducible determination, in each fracture, the post-operative 2-week images provided a template for all subsequent images, in which the same distance (mm) from the locking screw, proximal and distal to the defect region, was measured. Moreover, the selected defect region must enclose the entire radiologically distinguishable part of the defect. The surrounding bone refers to the healthy bone located around the defect, and this area should not exceed or fall below the defect region by more than 10%. The image analysis was blinded. The result was presented as a percentage of relative bone density (RBD), which was calculated using the following formula:

$$Percentage\;of\;relative\;bone\;density=\frac{RBD\;of\;fracture\;limb\times 100}{RBD\;of\;normal\;limb}$$

Because measuring the fracture gap (defect region) in the radius and tibia was limited, this study evaluated bone regeneration via the radiographic image of the ulna and fibula from each fracture.

### Statistical Analysis

The data are expressed as the mean and standard error (SE). An independent variable for all models was the interaction between groups and times, with both treated as categorical variables. For the model of CRP levels and CMPS-SF scores, times included days 1, 3, and 7, while the percentage of relative bone density was analyzed separately within the groups at 14 days, 1 month, and 2 months. The time factors of lameness and weight-bearing scores were categorized into 1, 3, 7, and 14 days, as well as at 1 month and 2 months. According to Norman [35], the use of the parametric method for ordinal data requires treating all ordinal variables as continuous. Therefore, all ordinal variables, including CMPS-SF, lameness, and weight-bearing scores, were treated as continuous data. Regarding the data collected from the same dogs, repeated-measures analyses were performed using a generalized linear mixed model (Proc mixed, SAS version 9.00, 2004) to identify group differences. Data from each patient were nested and analyzed under the Type 1 autocorrelation structure. The least-squares means were calculated and compared in both ways: (1) compare between groups at the same time, and (2) compare among times within the same group. The significance level was defined as *p* < 0.05. Effect sizes for the primary outcomes were calculated using Cohen’s *d*. For the group-by-time interaction, we calculated the standardized mean difference change using the pooled baseline standard deviation as the standardizer, as recommended by Morris [36]. Values of 0.2, 0.5, and 0.8 were used to represent small, medium, and large effects, respectively [37].

## 3. Results

In total, 12 dogs were included in the study, and their information and fracture details are shown in Table 1. Most dogs were small breeds, including Pomeranian (*n* = 7) and Chihuahua (*n* = 1). The average values of age (10.17 ± 1.17 years) and weight (5.54 ± 1.96 kg) in the A-PRF group were not different from those of the control (8.17 ± 1.74 years, 5.28 ± 1.67 kg), with significance at *p* = 0.37 and *p* = 0.95, respectively. All fractures were confirmed as aseptic with the same bone segment in both groups.

**Figure 1:**
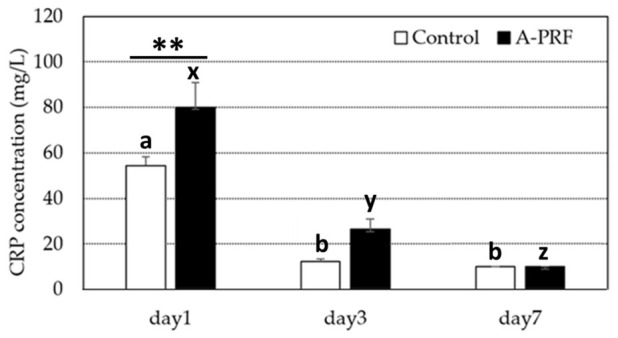
Serum CRP concentration (mean ± SD) of dogs in the control group compared with the A-PRF group on days 1, 3, and 7 after the operation. ^a,b; x,y,z^ are different letters indicating significant differences at *p* < 0.05 among times within the control and A-PRF, respectively. ** indicating significant differences between the control and A-PRF at the specified time.

All CRP levels at day 7 were <10 mg/L, and those at 10 mg/L were defined as elevated. The means (ranges) of the CRP from the control group and the A-PRF-treated group were 54.53 mg/L (42.3–69) and 80.15 mg/L (58.9–132.6) at day 1, 12.31 mg/L (10.6–18) and 26.47 mg/L (13–42.5) at day 3, and <10 mg/L and <10 mg/L at day 7, respectively. A comparison of the CRP level (means and SEs) between the control and A-PRF at 1, 3, and 7 days is shown in Figure 1. CRP levels in both groups were highest on day 1 and decreased significantly on day 3. At day 7, the CRP level in A-PRF decreased significantly compared with day 3, whereas the control showed no significant decrease. In the comparison between the control and A-PRF, the CRP from A-PRF was significantly higher than that from the control on day 1. On days 3 and 7, no significant difference in CRP levels was observed between the control and A-PRF groups.

**Figure 2:**
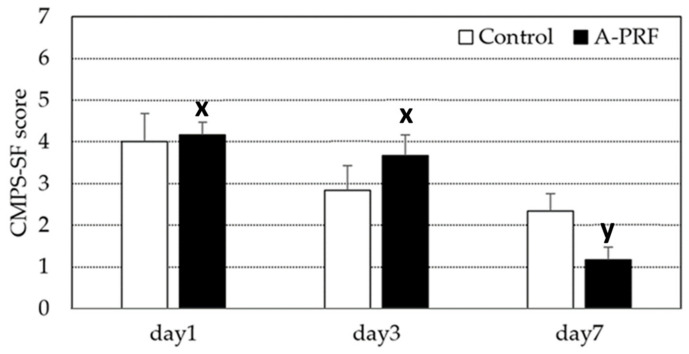
Pain score (mean ± SD) of dogs in the control group compared with the A-PRF group, and Glasgow Composite Measure Pain Scales (CMPS-SF) at days 1, 3, and 7 after the operation. ^x,y^ different letters indicating significant differences among times within the A-PRF group.

The pain score for A-PRF compared to the control is shown in Figure 2. The score level recommended for providing analgesia is ≥ 5. In our study, the mean pain score in both the A-PRF and control groups was below five throughout the observation period and decreased over time. Compared within the group, A-PRF showed a significant decrease in the pain score on day 7 compared to day 3, whereas this significant decrease was not observed in the control group. No significant difference in pain scores between A-PRF and the control was observed during the study period.

All groups experienced a spontaneous decrease in their lameness and weight-bearing scores during the study period (Table 2). The lameness score and the weight-bearing score in the A-PRF group showed no significant difference compared to the control on days 1, 3, and 7 and on days 1 and 3, respectively. On day 7 after surgery, A-PRF showed a significantly lower weight-bearing score than the control, followed by significantly lower lameness scores at day 14 and 1 month. There was no significant difference in lameness scores between A-PRF and the control at 2 months. From day 14 to the end of the study, A-PRF showed no significant difference in weight-bearing scores compared to the control group. All dogs in the A-PRF group showed no lameness (score = 0) and had normal weight-bearing scores (score = 2) at 1 month post-surgery, while this was observed in the control group at 2 months.

**Figure 3:**
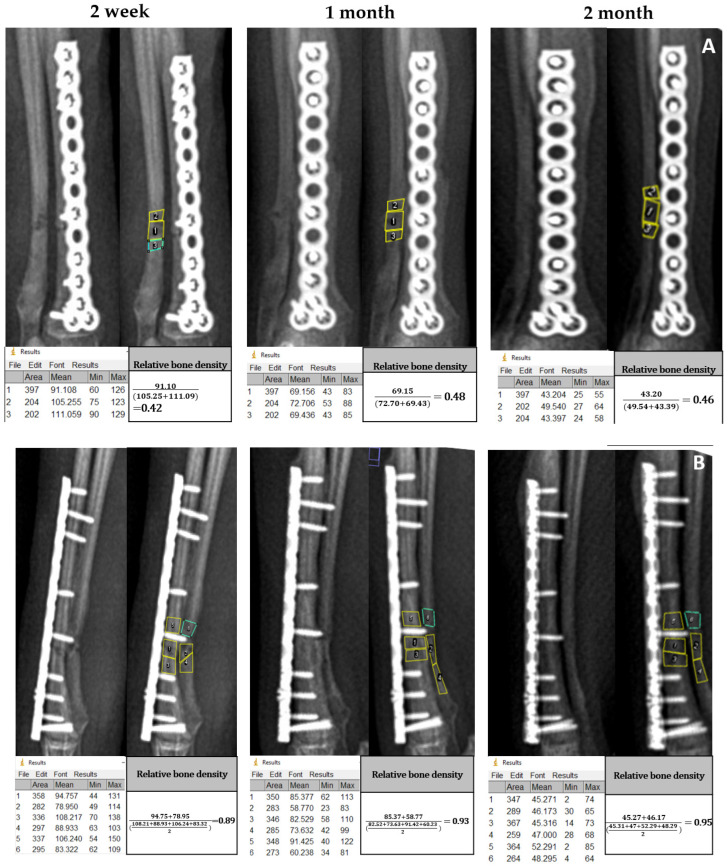

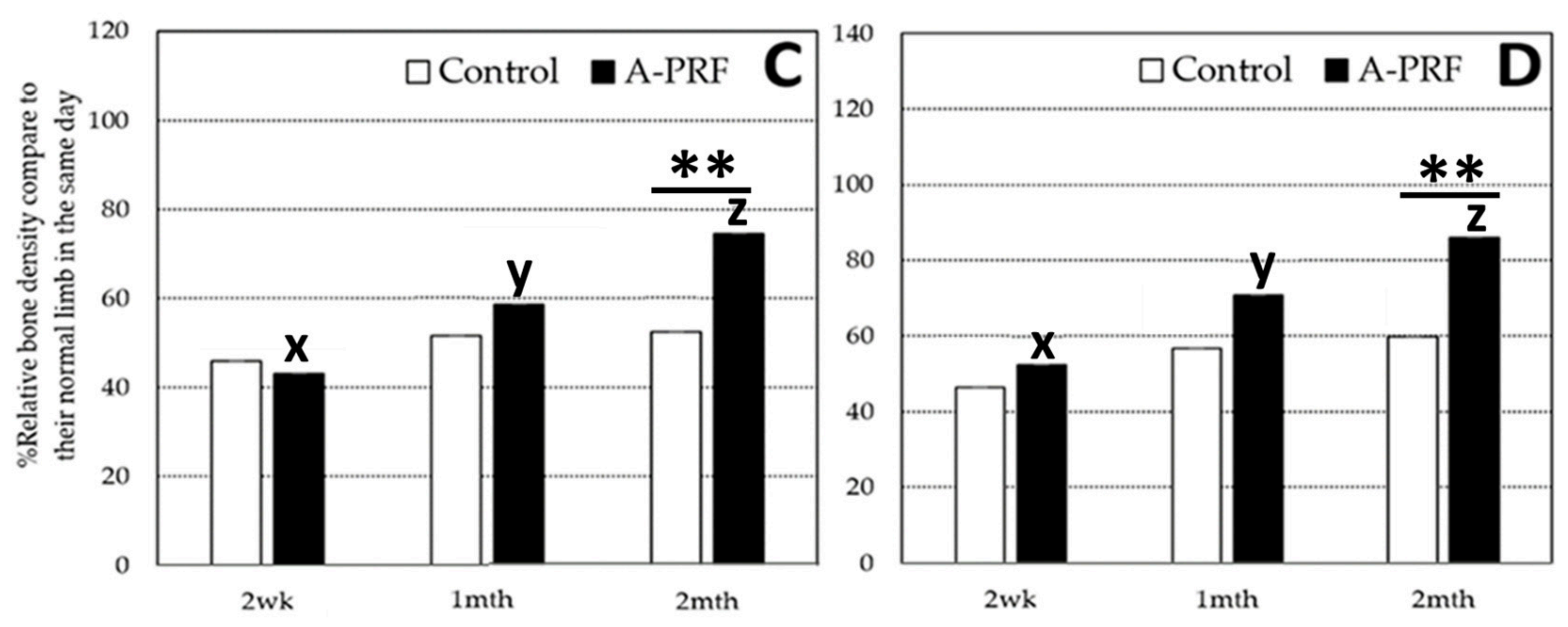
Cranio-caudal view (**A**) and mediolateral view (**B**) of post-operative radiographic images at 2 weeks, 1 month, and 2 months of radius–ulna fracture treated with A-PRF were measured using the mean gray value using ImageJ software. Mean gray values of the defect region and surrounding region are indicated by numbers 1 and 2–3 in (**A**) and 1–2 and 3–6 in (**B**), respectively. Relative bone density is calculated and shown. As shown in the area column, the area of the surrounding region does not exceed or fall below the defect region by more than 10 percent. The lower series figure indicates the percentage of relative bone density of A-PRF compared to control at 2 weeks, 1 month, and 2 months for the cranio-caudal view (**C**) and mediolateral view (**D**). ^x,y,z^ are different letters indicating significant differences among times within the A-PRF group. ** indicating significant differences between the control and A-PRF at the specified time.

Indirect bone healing was evaluated using the relative bone density (%) value calculated from radiographic images in the craniocaudal view and the mediolateral view. Overall relative bone density (%) ranged from 22.51 at D14 to 112.38 at 2 months after operation. For the craniocaudal radiographic view, the mean and their ranges of relative bone density percentages from the A-PRF and control group were 43.16 (36.05–60.50) and 45.93 (22.5–65.69) at 2 weeks, 58.60 (34.68–94.05) and 51.56 (21.48–93.64) at 1 month, and 74.38 (46.48–103.97) and 52.41 (21.52–103.65) at 2 months, respectively. For the mediolateral radiographic view, the mean and their ranges of relative bone density percentages from the A-PRF and control group were 52.26 (31.83–89.83) and 46.30 (36.86–59.29) at 2 weeks, 70.58 (37.84–93.65) and 56.61 (47.85–85.84) at 1 month, and 85.96 (50.57–112.37) and 59.73 (44.67–107.45) at 2 months, respectively. Representative time-lapse radiographic images of the A-PRF group in craniocaudal and mediolateral views at 2 weeks, 1 month, and 2 months post-operation are shown in Figure 3A,B. The results demonstrate that A-PRF increases relative bone density during the study period, indicating bone-healing activity. The relative bone density of A-PRF compared to the control at 2 weeks, 1 month, and 2 months is shown in Figure 3C,D. At 2 weeks and 1 month, there was no significant difference in the relative bone density percentage between the control and A-PRF for both the craniocaudal view and mediolateral view in the radiographic image. A-PRF showed a significantly higher relative bone density at 2 months than the control in both radiographic views. In comparison, within the A-PRF group, the percentage of relative bone density at 1 month and 2 months was significantly higher than at 2 weeks for both views.

The effect sizes for all dependent variables are shown in Table 3. Effect sizes of CRP, CMPS score, cranio-caudal relative bone density, and mediolateral relative bone density were 0.392, 0.428, 0.425, and 0.341, respectively, indicating that the impact of A-PRF on all parameters was close to the medium effect, meaning that supplementation with A-PRF had some treatment effect. However, there is still significant overlap between patients who receive A-PRF and those who do not. Effect sizes less than 0.1 for both lameness and weight-bearing scores indicated no difference between the standard treatment and A-PRF supplementation. Individual measurements and raw data of CRP level, limb functionality scores, and relative bone density are provided in Supplementary Materials.

## 4. Discussion

The use of A-PRF, an advanced PRF with more growth factors [38], was further evaluated in this study. Specifically, its effect on bone healing in appendicular fractures was examined, thereby providing direct clinical benefits. In this study, analyzing a clinical series of 12 dogs with appendicular fractures across different breeds provides a focused, real-world look at orthopedic trauma. Such a small, diverse sample size offers valuable qualitative insights, but it might face significant statistical hurdles. Mixing breeds introduces variability in bone density, muscle mass, and activity levels, and the quality of A-PRF, especially in terms of growth factors, also differed among dogs. These “confounders” make it difficult to determine if a successful healing process was due to the surgical technique or the dog’s natural physiology. To minimize confounding, before-and-after comparisons within the same subject were performed using a repeated-measures linear model, which relaxes the assumptions of the general linear model using the relative data. Regarding the evaluation of bone healing, lameness, and weight-bearing score, these may be useful for describing limb function, but it is important to keep in mind that these measurements are subjective. Moreover, the bone density results are sensitive to the region of interest selected. Therefore, given the limited sample size and qualitative and quantitative evaluation of parameters, the study results must be interpreted with caution.

In this study, locking compression plates and screws provided internal fixation, contributing to primary healing of the radius and tibia, while the ulna and fibula underwent secondary healing. Platelet-derived growth factors are involved in several stages of bone repair by producing numerous growth factors [39], and the fibrin mesh structure of A-PRF can facilitate the sustained release of these factors [40,41]. Therefore, the fracture healing process can be encouraged. Warin and colleagues [29] used the same technique for A-PRF preparation and found elevated levels of growth factors, including TGF-β1, VEGF-A, and PDGF-BB. However, the in vitro study might not absolutely explain their biological characterization on bone healing. The mechanism of A-PRF and its hematological parameters and growth factors release on bone healing must be further investigated.

In the first stage, an acute inflammatory response removes necrotic tissue and initiates angiogenesis [42]. C-reactive protein is one of the acute-phase proteins present in serum during the acute-phase response and is considered an early, nonspecific defense mechanism against local tissue injury and bacterial infection [43,44]. In veterinary medicine, healthy dogs exhibit low CRP concentrations of <10–25 mg/L [45]. After surgery, both groups had CRP levels higher than 50 mg/L, indicating local tissue injury after operation. Post-operative CRP elevation is expected after orthopedic surgery [46,47]. The higher CRP in the A-PRF group at day 1 might indicate greater tissue injury, likely due to the extended surgical time and increased pressure at the surgical wound when A-PRF was added. In general, excessive inflammation could impair bone healing and increase healing time [44]. However, this high level of inflammation from A-PRF did not worsen the patient’s condition, as our study found that pain, lameness, and weight-bearing scores were not significantly higher in the A-PRF group than in the control group throughout the study period. Moreover, the CRP level in the A-PRF group decreased significantly at each subsequent measurement, indicating resolution from inflammation [48]. The accelerated wound healing of A-PRF might be related to the chemotaxis of acute inflammatory cells induced by platelet-derived growth factors (PDGFs) and transforming growth factors (TGF-β) [49], and it may also be related to angiogenesis induced by vascular endothelial growth factor (VEGF) [43].

Following the inflammatory stage, around days 5 to 30, osteoblasts and chondroblasts increase and begin to form a soft callus, which then undergoes further development to form a hard callus. PRF was reported to promote the proliferation of this cell [20,25]. At this time, our study found that A-PRF showed significantly better weight-bearing (day 7) and lameness scores (days 14 and 30) than the control group. This, in turn, generates a transmission force that stimulates osteo-induction [50]. Lameness leads to a loss of balance when maintaining a static position, as evidenced by the patient shifting weight-bearing from the painful limb to the healthier contralateral limb to alleviate discomfort [51]. Therefore, lameness and weight-bearing scores could be predictive tools for assessing bone healing. For the final remodeling step, the bone becomes more rigid and stable, a change that lasts for months to years.

While computed tomography (CT) offers a more precise volumetric measurement of bone mineral density via Hounsfield units, it was not utilized in this study due to the potential for significant metal artifacts caused by the internal fixation hardware. Such artifacts can create “streaking” shadows that obscure the fracture callus, potentially leading to inaccurate density readings. Therefore, digital radiography was chosen as a reliable and accessible method to longitudinally monitor relative bone healing. Our study found that A-PRF showed significantly higher bone density than the control at 2 months, whereas no significant difference was observed at 2 weeks or 1 month. This finding was supported by a previous study of PRF in a rabbit bone defect [46], which reported no significant increase in bone volume in PRF compared to the control before 1.5 months, but significant differences were observed thereafter. While both groups start at similar levels at the 2-week mark, the A-PRF group shows an accelerated, linear increase in relative bone density, reaching statistical significance by the 1- and 2-month marks. The significant increase in relative bone density in the A-PRF group from two weeks to two months, compared with the stagnant control group, is largely due to the A-PRF matrix’s unique structure and sustained growth factor release. A-PRF is generated at lower centrifugation speeds, preserving a higher concentration of leukocytes and creating a porous fibrin scaffold that enhances osteoblast migration and angiogenesis [22,51]. This scaffold enables the sustained release of vital growth factors, such as TGF-β and VEGF, which are essential for bone mineralization and remodeling, particularly at two months [27,52]. Consequently, while the control group lacks the sustained signaling required for accelerated densification, the A-PRF group benefits from a continuous “bio-reservoir”, resulting in significantly improved recovery levels.

The pain level associated with bone fractures is reported to be severe. It is crucial to ensure adequate pain relief to shorten recovery time. According to WASAVA guidelines for the recognition of pain, the post-surgical pain scoring system is the Glasgow Composite Measure Pain Scale in its short form (CMPS-SF), which is primarily based on behaviors [53]. In our study, the mean pain score of both A-PRF and the control was less than five during the observation because all patients received multi-modal analgesia, including opioids in premedication, local anesthesia, and NSAIDs. A previous study by Reeshma and colleagues [54] reported pain relief with PRF in patients undergoing tooth extraction. Although there was no significant difference in pain scores between the A-PRF and control groups, the pain score in the A-PRF group decreased over time, with a significant difference observed on day 7 compared to day 3.

## 5. Conclusions

In conclusion, A-PRF demonstrated promising bone-healing results, as evidenced by significantly higher bone density of the ulna and fibula at 1 month compared with the baseline and at 2 months compared with the control group. Applying A-PRF reduced lameness scores more quickly than in the control group at 14 days. Due to the limited sample size in this study, further larger clinical studies would be required to validate these results. In addition, both groups achieved full functional recovery within two months, raising important questions about the clinical relevance of the modest early differences observed. The authors acknowledge that this model may represent a favorable healing environment and that the design may also underestimate or fail to reveal the potential benefits of A-PRF in biologically compromised or high-risk fractures. The current conclusions might be reframed to reflect this limitation in future studies.

## Figures and Tables

**Table 1:** Data of the dog with traumatic bone fracture treatment, including the A-PRF group and control.

Dog	Age (Month)	Sex	Weight (kg)	Breed	Bone Fracture Type	Bone Segment
**A-PRF group**						
1	12	male	4.6	Pomeranian	distal-diaphyseal, short oblique	radius–ulna
2	6	male	7.6	French bulldog	proximal-diaphyseal, transverse	tibia–fibula
3	12	female	2.2	Pomeranian	distal-diaphyseal, short oblique	radius–ulna
4	7	male	2.7	Pomeranian	distal-diaphyseal, short oblique	radius–ulna
5	12	male	1.85	Pomeranian	distal –diaphyseal, short oblique	radius–ulna
6	12	female	14.3	Thai	mid-diaphyseal, short oblique	tibia–fibula
**Control group**						
7	4	male	1.5	Pomeranian	distal-diaphyseal, short oblique	radius–ulna
8	6	female	12	Thai	distal-diaphyseal, short oblique	radius–ulna
9	5	female	1.6	Pomeranian	mid-diaphyseal, short oblique	radius–ulna
10	13	female	3	Chihuahua	distal-diaphyseal, short oblique	radius–ulna
11	7	male	7.5	Thai	mid-diaphyseal, short oblique	tibia–fibula
12	14	female	6.1	Pomeranian	proximal-diaphyseal, short oblique	tibia–fibula

**Table 2:** Lameness score and weight-bearing score (mean ± SD) of the A-PRF group compared with the control group at 1, 3, 7 days, 2 weeks, 1 month, and 2 months post-operation.

	Lameness Score		Weight-Bearing Score	
Time	Control	A-PRF	Control	A-PRF
**Day 1**	4 ± 0 ^a^	4 ± 0 ^w^	9.8 ± 0.1 ^a^	9.1 ± 0.3 ^w^
**Day 3**	3.5 ± 0.3 ^a^	3.5 ± 0.2 ^w^	9 ± 0.4 ^a^	8.3 ± 0.3 ^w^
**Day 7**	2.8 ± 0.3 ^a^	2.3 ± 0.4 ^x^	8 ± 0.5 ^b^**	6 ± 0.9 ^x^**
**Day 14**	1.8 ± 0.3 ^b^**	0.8 ± 0.3 ^y^**	4.5 ± 0.6 ^c^	3.5 ± 0.8 ^y^
**1 month**	0.6 ± 0.2 ^c^**	0 ± 0 ^z^**	3.3 ± 0.4 ^d^	2 ± 0 ^z^
**2 months**	0 ± 0 ^c^	0 ± 0 ^z^	2 ± 0 ^e^	2 ± 0 ^z^

^a,b,c,d,e; w,x,y,z^: different letters indicate significant differences at *p* < 0.05 among times within the control and A-PRF, respectively. ** indicating significant differences at *p* < 0.05 between the control and A-PRF at the specified time.

**Table 3:** Effect size of time and treatment groups on outcome variables using Cohen’s *d* value using the pooled baseline standard deviation as the standardizer [36].

Outcome Variable	F-Statistic	*p*-Value	Cohen’s *d*
CRP	47.6	<0.01	0.392
CMPS score	5.74	0.0022	0.428
Lameness score	35.72	0.01	<0.1
Weight-bearing score	31.78	<0.001	<0.1
Cranio-caudal relative bone density	3.47	0.02	0.425
Mediolateral relative bone density	4.69	0.005	0.341

## Data Availability

Data will be available for all readers upon request.

## Supplementary Materials

The following supporting information can be downloaded at https://www.mdpi.com/article/10.3390/ani16081276/s1, Table S1: Serum CRP concentration (mg/L) from dogs with traumatic bone fracture; Table S2: Pain score of dogs with traumatic bone fracture evaluated using Glasgow Composite Measure Pain Scales (CMPS-SF) at day 1, 3, and 7 post-operation; Table S3: Lameness score and weight-bearing score of dogs with traumatic bone fracture at days 1, 3, and 7, 2 weeks, 1 month, and 2 months post-operation; Table S4: Relative bone density (%) of bone fracture in cranio-caudal view and mediolateral view at 2 week, 1 month, and 2 months post-operation of dog treatment with A-PRF and control.

## References

[1] Johnson J.A., Austin C., Breur G.J. (1994). Incidence of canine appendicular musculoskeletal disorders in 16 veterinary teaching hospitals from 1980 through 1989. Vet. Comp. Orthop. Traumatol..

[2] Phillips I.R. (1979). A survey of bone fractures in the dog and cat. J. Small Anim. Pract..

[3] Tsiridis E., Upadhyay N., Giannoudis P. (2007). Molecular aspects of fracture healing: Which are the important molecules?. Injury.

[4] Cheal E.J., Hayes W.C., White A.A., Perren S.M. (1985). Stress analysis of compression plate fixation and its effects on long bone remodeling. J. Biomech..

[5] Aro H.T., Chao E.Y.S. (1993). Biomechanics and biology of fracture repair under external fixation. Hand Clin..

[6] Duan Z.W., Lu H. (2021). Effect of Mechanical Strain on Cells Involved in Fracture Healing. Orthop. Surg..

[7] Hausman M.R., Schaffler M.B., Majeska R.J. (2001). Prevention of fracture healing in rats by an inhibitor of angiogenesis. Bone.

[8] Garofolo S., Pozzi A. (2013). Effect of plating technique on periosteal vasculature of the radius in dogs: A cadaveric study. Vet. Surg..

[9] Matres-Lorenzo L., Diop A., Maurel N., Boucton M.C., Bernard F., Bernardé A. (2016). Biomechanical Comparison of Locking Compression Plate and Limited Contact Dynamic Compression Plate Combined with an Intramedullary Rod in a Canine Femoral Fracture-Gap Model. Vet. Surg..

[10] Menghini T.L., Shriwise G., Muir P. (2023). Fracture Healing in 37 Dogs and Cats with Implant Failure after Surgery (2013–2018). Animal.

[11] Vallefuoco R., Le Pommellet H., Savin A., Decambron A., Manassero M., Viateau V., Gauthier O., Fayolle P. (2016). Complications of appendicular fracture repair in cats and small dogs using locking compression plates. Vet. Comp. Orthop. Traumatol..

[12] Ganse B. (2024). Methods to accelerate fracture healing—A narrative review from a clinical perspective. Front. Immunol..

[13] Oprea W.E., Karp J.M., Hosseini M.M., Davies J.E. (2003). Effect of platelet releasate on bone cell migration and recruitment in vitro. J. Craniofac. Surg..

[14] Wu M., Chen G., Li Y.-P. (2016). TGF-β and BMP signaling in osteoblast, skeletal development, and bone formation, homeostasis and disease. Bone Res..

[15] Canapp S.O., Leasure C.S., Cox C., Ibrahim V., Carr B.J. (2016). Partial cranial cruciate ligament tears treated with stem cell and platelet-rich plasma combination therapy in 36 dogs: A retrospective study. Front. Vet. Sci..

[16] Franklin S.P., Burke E.E., Holmes S.P. (2017). The effect of platelet-rich plasma on osseous healing in dogs undergoing high tibial osteotomy. PLoS ONE.

[17] Zhang Y., Xing F., Luo R., Duan X. (2021). Platelet-Rich Plasma for Bone Fracture Treatment: A Systematic Review of Current Evidence in Preclinical and Clinical Studies. Front. Med..

[18] Warin R., Vongchan P., Suriyasathaporn W., Hall D.C., Boripun R., Suriyasathaporn W. (2023). In Vitro Antimicrobial Properties and Their Mechanisms in Relation to Reactive Oxygen Species of Canine Platelet-Rich Fibrin. Animals.

[19] Idulhaq M., Mudigdo A., Utomo P., Wasita B., Warman F.I. (2022). Platelet-rich fibrin as a tissue engineering material in accelerate bone healing in rat bone defects: A systematic review and meta-analysis. Ann. Med. Surg..

[20] Wang X., Zhang Y., Choukroun J., Ghanaati S., Miron R.J. (2018). Effects of an injectable platelet-rich fibrin on osteoblast behavior and bone tissue formation in comparison to platelet-rich plasma. Platelets.

[21] Dohan Ehrenfest D.M., Rasmusson L., Albrektsson T. (2009). Classification of platelet concentrates: From pure platelet-rich plasma (P-PRP) to leucocyte- and platelet-rich fibrin (L-PRF). Trends Biotechnol..

[22] Kobayashi E., Flückiger L., Fujioka-Kobayashi M., Sawada K., Sculean A., Schaller B., Miron R.J. (2016). Comparative release of growth factors from PRP, PRF, and advanced-PRF. Clin. Oral Investig..

[23] Jia K., You J., Zhu Y., Li M., Chen S., Ren S., Chen S., Zhang J., Wang H., Zhou Y. (2024). Platelet-rich fibrin as an autologous biomaterial for bone regeneration: Mechanisms, applications, optimization. Front. Bioeng. Biotechnol..

[24] Kazemi D., Fakhrjou A. (2015). Leukocyte and Platelet Rich Plasma (L-PRP) Versus Leukocyte and Platelet Rich Fibrin (L-PRF) For Articular Cartilage Repair of the Knee: A Comparative Evaluation in an Animal Model. Iran. Red Crescent Med. J..

[25] Kazemi D., Fakhrjou A., Dizaji V.M., Alishahi M.K. (2014). Effect of autologous platelet rich fibrin on the healing of experimental articular cartilage defects of the knee in an animal model. Biomed. Res. Int..

[26] Hoang-Kim A., Miclau T., Goldhahn J., Nijman T.H., Poolman R.W. (2014). Patient-important outcome for the assessment of fracture repair. Injury.

[27] Choukroun J., Ghanaati S. (2018). Reduction of relative centrifugation force within injectable platelet-rich-fibrin (PRF) concentrates advances patients’ own inflammatory cells, platelets and growth factors: The first introduction to the low speed centrifugation concept. Eur. J. Trauma Emerg. Surg..

[28] McMillan M., Brearley J. (2013). Assessment of the variation in American Society of Anesthesiologists [corrected] Physical Status Classification assignment in small animal anaesthesia. Vet. Anaesth. Analg..

[29] Warin R., Vongchan P., Suriyasathaporn W., Boripun R., Suriyasathaporn W. (2022). In Vitro Assessment of Lyophilized Advanced Platelet-Rich Fibrin from Dogs in Promotion of Growth Factor Release and Wound Healing. Vet. Sci..

[30] Schatzker J. (1995). Changes in the AO/ASIF principles and methods. Injury.

[31] Reid J., Nolan A.M., Hughes J.M.L., Lascelles D., Pawson P., Scott E.M. (2007). Development of the short-form Glasgow Composite Measure Pain Scale (CMPS-SF) and derivation of an analgesic intervention score. Anim. Welf..

[32] Wangdee C., Theyse L.F., Techakumphu M., Soontornvipart K., Hazewinkel H.A. (2013). Evaluation of surgical treatment of medial patellar luxation in Pomeranian dogs. Vet. Comp. Orthop. Traumatol..

[33] Monk M.L., Preston C.A., McGowan C.M. (2006). Effects of early intensive postoperative physiotherapy on limb function after tibial plateau leveling osteotomy in dogs with deficiency of the cranial cruciate ligament. Am. J. Vet. Res..

[34] Geiger M., Blem G., Ludwig A. (2016). Evaluation of ImageJ for relative bone density measurement and clinical application. J. Oral Health Craniofacial Sci..

[35] Norman G. (2010). Likert scales, levels of measurement and the “laws” of statistics. Adv. Health Sci. Educ. Theory Pract..

[36] Morris S. (2008). Estimating Effect Sizes From Pretest-Posttest-Control Group Designs. Organ. Res. Methods.

[37] Cohen J. (1988). Statistical Power Analysis for the Behavioral Sciences.

[38] Fujioka-Kobayashi M., Miron R.J., Hernandez M., Kandalam U., Zhang Y., Choukroun J. (2017). Optimized Platelet-Rich Fibrin With the Low-Speed Concept: Growth Factor Release, Biocompatibility, and Cellular Response. J. Periodontol..

[39] Sheen J.R., Mabrouk A. Fracture Healing Overview. https://www.ncbi.nlm.nih.gov/books/NBK551678/.

[40] Choukroun J., Diss A., Simonpieri A., Girard M.O., Schoeffler C., Dohan S.L., Dohan A.J., Mouhyi J., Dohan D.M. (2006). Platelet-rich fibrin (PRF): A second-generation platelet concentrate. Part IV: Clinical effects on tissue healing. Oral Surg. Oral Med. Oral Pathol. Oral Radiol. Endod..

[41] Dohan Ehrenfest D.M., Bielecki T., Jimbo R., Barbé G., Del Corso M., Inchingolo F., Sammartino G. (2012). Do the fibrin architecture and leukocyte content influence the growth factor release of platelet concentrates? An evidence-based answer comparing a pure platelet-rich plasma (P-PRP) gel and a leukocyte- and platelet-rich fibrin (L-PRF). Curr. Pharm. Biotechnol..

[42] ElHawary H., Baradaran A., Abi-Rafeh J., Vorstenbosch J., Xu L., Efanov J.I. (2021). Bone Healing and Inflammation: Principles of Fracture and Repair. Semin. Plast. Surg..

[43] Hankenson K.D., Dishowitz M., Gray C., Schenker M. (2011). Angiogenesis in bone regeneration. Injury.

[44] Claes L., Recknagel S., Ignatius A. (2012). Fracture healing under healthy and inflammatory conditions. Nat. Rev. Rheumatol..

[45] Buchner H.H., Obermüller S., Scheidl M. (2001). Body centre of mass movement in the lame horse. Equine Vet. J. Suppl..

[46] Macleod C.M., Avery O.T. (1941). The occurrence during acute infections of a protein not normally present in the blood: Iii. immunological properties of the c-reactive protein and its differentiation from normal blood proteins. J. Exp. Med..

[47] Murtaugh M.P., Baarsch M.J., Zhou Y., Scamurra R.W., Lin G. (1996). Inflammatory cytokines in animal health and disease. Vet. Immunol. Immunopathol..

[48] Gewurz H., Mold C., Siegel J., Fiedel B. (1982). C-reactive protein and the acute phase response. Adv. Intern. Med..

[49] Martínez C.E., Smith P.C., Palma Alvarado V.A. (2015). The influence of platelet-derived products on angiogenesis and tissue repair: A concise update. Front. Physiol..

[50] Meadows T.H., Bronk J.T., Chao Y.S., Kelly P.J. (1990). Effect of weight-bearing on healing of cortical defects in the canine tibia. J. Bone Jt. Surg. Am..

[51] Ghanaati S., Booms P., Orlowska A., Kubesch A., Lorenz J., Rutkowski J., Landes C., Sader R., Kirkpatrick C., Choukroun J. (2014). Advanced platelet-rich fibrin: A new concept for cell-based tissue engineering by means of inflammatory cells. J. Oral Implantol..

[52] Miron R.J., Zhang Y. (2018). Autologous liquid platelet rich fibrin: A novel drug delivery system. Acta Biomater..

[53] Monteiro B.P., Lascelles B.D.X., Murrell J., Robertson S., Steagall P.V.M., Wright B. (2023). 2022 WSAVA guidelines for the recognition, assessment and treatment of pain. J. Small Anim. Pract..

[54] Reeshma S., Dain C.P. (2021). Comparison of platelet-rich fibrin with zinc oxide eugenol in the relief of pain in alveolar osteitis. Health Sci. Rep..

