# Supplementary material for: Effects of Advanced Platelet-Rich Fibrin on Bone Healing in the Treatment of Canine Appendicular Fractures
Source: Animals (Basel). 2026 Apr 21;16(8):1276. doi: 10.3390/ani16081276 (PMC13113820; doi:10.3390/ani16081276)
Supplement: Supplementary file 1 [file animals-16-01276-s001.zip › Supplementary Materials 1.pdf]

Table S1. Serum CRP concentration (mg/L) from dogs with traumatic bone fracture.

Dog	Day post-operation		
	**1**	**3**	**7**
**A-PRF group**			
1	66.2	21.9	<10
2	78.2	34.2	<10
3	72.1	13	<10
4	132.6	42.5	<10
5	58.9	17.7	<10
6	72.9	29.5	<10
**Control group**			
7	58.7	11.2	<10
8	55.3	11.9	<10
9	42.3	10.8	<10
10	46.8	11.4	<10
11	69	18	<10
12	55.1	10.6	<10
