# Supplementary material for: Effects of Advanced Platelet-Rich Fibrin on Bone Healing in the Treatment of Canine Appendicular Fractures
Source: Animals (Basel). 2026 Apr 21;16(8):1276. doi: 10.3390/ani16081276 (PMC13113820; doi:10.3390/ani16081276)
Supplement: Supplementary file 1 [file animals-16-01276-s001.zip › Supplementary Materials 2.pdf]

Table S2. Pain score of dogs with traumatic bone fracture evaluated using Glasgow composite measure pain scales (CMPS-SF) at day1,3, and 7 post-operation.

**Dog**	**Day post-operation**		
	**day1**	**day3**	**day7**
**A-PRF**			
1	4	6	2
3	3	3	1
4	5	3	1
5	5	3	0
7	4	3	1
8	4	4	2
**Control**			
6	5	2	2
2	6	1	1
9	2	2	4
10	2	3	2
11	4	4	2
12	5	5	3
