# Supplementary material for: Effects of Advanced Platelet-Rich Fibrin on Bone Healing in the Treatment of Canine Appendicular Fractures
Source: Animals (Basel). 2026 Apr 21;16(8):1276. doi: 10.3390/ani16081276 (PMC13113820; doi:10.3390/ani16081276)
Supplement: Supplementary file 1 [file animals-16-01276-s001.zip › Supplementary Materials 3.pdf]

Table S3. Lameness score and weight-bearing score of dogs with traumatic bone fracture at 1,3,7 day, 2 week, 1month, and 2 months post-operation.

Dog	Lameness score						Weight-bearing score					
	**Day1**	**Day3**	**Day7**	**2wk**	**1mth**	**2mth**	**Day1**	**Day3**	**Day7**	**2wk**	**1mth**	**2mth**
**A-PRF**												
**1**	4	3	1	1	0	0	10	8	4	2	2	2
**2**	4	3	2	0	0	0	9	8	4	2	2	2
**3**	4	4	3	1	0	0	9	8	6	4	2	2
**4**	4	4	4	2	0	0	10	10	10	7	2	2
**5**	4	4	2	1	0	0	9	8	7	4	2	2
**6**	4	3	2	0	0	0	8	8	5	2	2	2
**Control**												
**7**	4	4	3	1	0	0	10	10	7	4	2	2
**8**	4	2	2	1	1	0	10	7	7	3	4	2
**9**	4	4	4	3	1	0	10	10	10	7	4	2
**10**	4	3	3	2	0	0	9	9	7	3	2	2
**11**	4	4	3	2	1	0	10	9	9	6	4	2
**12**	4	4	2	2	1	0	10	9	8	4	4	2
