# Supplementary material for: Effects of Advanced Platelet-Rich Fibrin on Bone Healing in the Treatment of Canine Appendicular Fractures
Source: Animals (Basel). 2026 Apr 21;16(8):1276. doi: 10.3390/ani16081276 (PMC13113820; doi:10.3390/ani16081276)
Supplement: Supplementary file 1 [file animals-16-01276-s001.zip › Supplementary Materials 4.pdf]

Table S4. Relative bone density (%) of bone fracture in cranio-caudal view and mediolateral view at 2 week, 1 month, and 2 month post-operation of dog treatment with A-PRF and control.

Dog	cranio-caudal image			Latero-lateral image		
	**2week**	**1month**	**2month**	**2week**	**1month**	**2month**
**A-PRF**						
**1**	39.24	36.52	71.77	42.32	37.85	50.57
**2**	40.54	48.48	79.07	31.83	57.76	89.83
**3**	36.06	34.69	48.70	45.77	58.09	79.81
**4**	42.12	48.65	46.49	89.84	93.66	94.81
**5**	60.51	94.06	96.28	71.92	87.97	88.40
**6**	40.51	89.22	103.98	31.88	88.18	112.38
**Control**						
**7**	65.69	93.65	103.66	49.23	85.85	107.45
**8**	61.63	49.56	47.09	44.36	47.86	49.78
**9**	43.98	50.21	48.36	37.51	49.98	44.67
**10**	35.58	48.11	45.61	36.87	47.86	49.60
**11**	46.21	46.41	48.26	50.54	49.21	50.19
**12**	22.51	21.49	21.53	59.30	58.94	56.70
